# Influence du comportement des accompagnants sur le vécu des patients admis pour hémorragies digestives hautes au CHU campus de Lomé (Togo)

**DOI:** 10.11604/pamj.2014.19.238.4545

**Published:** 2014-11-03

**Authors:** Aklesso Bagny, Angelique Dusabe, Oumboma Bouglouga, Mawuli Late Lawson-ananisoh, Yeba Laconi Kaaga, Mohaman Awalou Djibril, Kokou Mensah Soedje, Simliwa Kolou Dassa, Datouda Redah

**Affiliations:** 1 Service d'Hépato-Gastroentérologie, CHU - Campus de Lomé, Togo; 2 Service de Psychiatrie de Psychologie Médicale, CHU – Campus de Lomé, Togo; 3 Service de Médecine Interne et Urgence Médicale, CHU Sylvanus Olympio, Togo

**Keywords:** Hémorragie digestive haute, vécu, représentation, comportement, accompagnant, Upper gastrointestinal bleeding, life, representation, behavior, attendant

## Abstract

**Introduction:**

L'hémorragie digestive haute est une urgence, qui constitue souvent pour les patients un danger mortel suscitant inquiétude et agitation. Dans cet état, le patient dépend de ses accompagnants pour ses soins et pour honorer le traitement; mais souvent, il a été observé une discordance entre l'urgence et les comportements des accompagnants. Le but de cette étude était de décrire les facteurs socioéconomiques et psychologiques pouvant influencer les comportements des accompagnants des patients admis pour HDH, estimer l'indice de relation entre ces comportements et les facteurs associés d'une part et le vécu des patients admis pour HDH d'autre part.

**Méthodes:**

Il s'agit d'une étude prospective menée de Septembre 2010 à Juin 2011 (soit 10 mois). Nous avions utilisé l'entretien semi-dirigé et l'observation directe pour collecter nos données, ces dernières avaient été traitées par les méthodes statistiques et d'analyse de contenu.

**Résultats:**

Dans la présente étude, les comportements des accompagnants des patients admis pour HDH sont en majorité marqués par l'abandon (84%) et le manque de sollicitude (80,2%). Ces comportements sont souvent stimulés par les facteurs socioéconomiques tels que les difficultés économiques (83,2%), des conflits intrafamiliaux (85,1%) et des représentations (maladie incurable ou envoûtement) de la maladie par les accompagnants (73,3%) des cas. Quant aux patients, ils vivent ces comportements comme étant des menaces de mort ou des rejets (77,20%) et comme étant une dévalorisation ou une humiliation de la part de leurs accompagnants (70,30%). Les résultats confirment l'existence de lien significatif entre les comportements des accompagnants et les facteurs socio économiques, entre les comportements des accompagnants et des facteurs psychologiques, et entre le vécu des patients admis pour l'HDH et les comportements des accompagnants.

**Conclusion:**

Des études ultérieures devraient aborder les points non touchés dans cette étude. Certes, elle a apporté un plus dans la compréhension du vécu des patients admis pour HDH et des comportements de leurs accompagnants. Cependant, elle est loin d'avoir épuisé les différents contours de la problématique de la présente étude.

## Introduction

L'hémorragie digestive haute(HDH) est un saignement du tractus digestif supérieur en amont de l'angle de Treizt. Elle constitue une urgence médico-chirurgicale pouvant mettre en jeu le pronostic vital par un choc hémorragique [1]. La survenue de l'HDH constitue souvent pour les patients un danger mortel suscitant inquiétude et agitation. La méconnaissance de la maladie, la peur de s’être vidé de son sang, la peur de ne pas guérir, l'attitude et l'interprétation donnée par l'entourage proche des patients, l'exagération sur la quantité de sang extériorisé sont autant des facteurs qui altèrent la qualité de vie des patients. Dans cet état, le patient dépend entièrement de ses accompagnants pour honorer le traitement et souvent, il a été observé une discordance entre l'urgence et les comportements des accompagnants. Communément lorsqu'on parle d'accompagnant de patients, on pense directement à la famille c′est-à-dire à ceux sur qui on peut compter dans les moments difficiles surtout lors de la survenue d'une maladie brutale ou chronique [2]. Toutefois, on constate souvent que certains événements bouleversent ce petit groupe social: la discorde des couples, le deuil, les difficultés financières, la maladie surtout si elle est chronique et coûteuse. Le lien familial est souvent brisé par une pathologie où le patient est considéré lui-même comme la cause de sa maladie ou une pathologie qui suscite des conflits au sein de la famille [3]. Parfois, les patients hospitalisés pour HDH se retrouvent abandonnés par leur famille seul soutient dans les moments critiques de leur vie. La présente étude tente de décrire l'influence des comportements des accompagnants sur le vécu des patients admis pour hémorragie digestive haute afin d'apporter notre contribution à la prise en charge efficiente de ces patients.

## Méthodes

Nous avons utilisé la technique d’échantillonnage de tout venant. Nous avons donc considéré tous les cas présents pendant notre période d’étude et répondant aux critères de sélection notamment, tous les patients conscients ayant été hospitalisés dans le service de Gastro-Entérologie du CHU-Campus pour HDH et de leurs accompagnants. Pour cette étude nous avons utilisé la méthode de l'entretien semi structurée, et pour cela nous avons établi deux guides d'entretien, l'un pour les patients et l'autre à l'endroit des accompagnants. Les principaux thèmes abordés par les guides d'entretien pour cette étude portent sur l'identité des participants, le vécu du patient de sa maladie, le climat familial, la représentation de la maladie par l'accompagnant, la perception de l'accompagnant par le patient, le vécu du traitement par le patient et par les accompagnants. Les patients et leurs accompagnants étaient informés sur les objectifs de l’étude et avaient donné leur consentement éclairé. L'analyse statistique des données était effectuée à l'aide du logiciel de statistiques SPSS 18.0. Pour réaliser les statistiques inférentielles et pour calculer le Khi 2 et le coefficient de contingence C. Le P value des tests était comparé au seuil de 5%.

## Résultats

Pendant notre période d’étude, 67 patients et 101 accompagnants avaient répondu à nos critères d'inclusion. L’âge moyen de nos patients était de 39,4±13,9 ans avec une prédominance masculine (sex-ratio = 2,9). L’âge moyen des accompagnants était de 44,8±10,1 ans avec une prédominance féminine (sex-ratio = 15,8); les caractéristiques socio-démographiques des patients et des accompagnants sont données par le Tableau 1. Le vécu des patients par rapport aux comportements de leurs accompagnants était, un sentiment de menace de mort ou de rejet (77,2%), de dévalorisation (70,3%) ou encore de culpabilité (15,8%). Quant à la représentation de la maladie par les accompagnants, 73, 3% se représentaient l'HDH comme étant une maladie incurable ou un envoûtement; pour certain accompagnants (28,7%) les patients étaient eux-mêmes source de leur mal; et pour d'autres (12,9%) il s'agissait d'une maladie naturelle. Les comportements de ces accompagnants se manifestaient par l'abandon (83,2%), le manque de sollicitude (80,2%), l'agressivité (31,7%) ou encore par la sollicitude des soins (15,8%). La considération des facteurs socio-économiques montrait dans notre étude, qu'il existait une prédominance des conflits intrafamiliaux (85,10%), des difficultés financières (83,20%) et que seulement 13,9% avaient des moyens financiers pour assurer la prise en charge de leurs patients. L'analyse de la relation entre les comportements des accompagnants et les facteurs socio-économiques (Tableau 2) avait permit de trouver un coefficient de contingence égal à 0,54, montrant qu'il existait un lien fort entre les facteurs socio-économiques et les comportements des accompagnants. Les résultats montraient que 48% des patients abandonnés avaient des difficultés financières et 50% d'entre eux avaient des conflits intrafamiliaux. De même que les patients agressés, ceux qui manquaient de sollicitude avaient des difficultés financières dans 48,1%des cas et on notait des conflits intrafamiliaux dans 50%des cas.

**Tableau 1:** Données socio-démographiques des patients et des accompagnants

**Echantillon total des patients (N= 67)**	
**Age**	39,37 (E.T. = 13,93)
**Sexe**	
*Hommes*	50 (74,6%)
*Femmes*	17 (25,4%)
**Statut marital**	
*Célibataire*	22 (32,8%)
*Marié(e)*	34 (50,7%)
*Divorcé(e)*	7 (10,4%)
*Veuf (ve)*	4 (6%)
**Classe socio économique**	
*Elevée*	2 (3,0%)
*Moyenne*	20 (29,9%)
*Basse*	45 (67,2%)
**Echantillon total des accompagnants (N= 101)**	
***Age***	44,77 (E.T. = 10,14)
***Sexe***	
*Hommes*	6 (5,9%)
*Femmes*	95 (94,1%)
*Lien de parenté*	
*Parent proche*	50 (49,5%)
*Parent éloigné*	33 (32,7%)
*Personne inconnue*	18 (17,8%)

**Tableau 2:** Relation entre les comportements des accompagnants et les facteurs socioéconomiques

Facteurs socio-économiques	Difficulté économique (n/%)	Conflit intrafamiliaux (n/%)	Disponibilité des moyens financiers (n/%)	TOTAL (n/%)
Comportements de l'entourage				
Abandon	81 48,8%	83 50,0%	2 1,2%	166 100%
Agressivité	31 48,4%	32 50,0%	1 1,6%	64 100%
Manque de sollicitude	77 48,1%	80 50,0%	3 1,9%	160 100%
Sollicitude	3 18,8%	2 12,5%	11 68,8%	16 100%
TOTAL	192 47,3%	197 48,5%	17 4,2%	406 100%

C= 0,326 p < 0,0001 X^2^=173,23

Ces résultats confirmaient l'hypothèse selon laquelle les facteurs socio-économiques influencent les comportements des accompagnants des patients admis pour HDH. Nos résultats montraient qu'il existait une relation moyenne (C = 0,27 p <0,0001) entre la représentation de la maladie par les accompagnants et leurs comportements (Tableau 3), On notait alors chez les patients abandonnés, agressés ou ceux qui manquaient de sollicitude; que leurs accompagnants se représentaient leur maladie comme étant incurable et/ ou lié à l'envoûtement respectivement dans 67,3%, 61,5% et 65,3% des cas; confirmant ainsi notre hypothèse selon laquelle les facteurs psychologiques tels que les représentations influençaient les comportements des accompagnants des patients admis pour HDH. Pour ce qui concerne le vécu du patient (Tableau 4) et les comportements de leurs accompagnants, on notait l'existence d'une relation forte (C = 0,62 p<0,0001). Les patients qui bénéficiaient de sollicitude de la part de leurs accompagnants vivaient leur maladie avec assurance dans 88,2% des cas; Les patients abandonnés vivaient cette situation comme une menace de mort dans 42%des cas, ou comme une dévalorisation dans 41,9% et avaient un sentiment de culpabilité dans 44,1% des cas; Ceux qui manquaient de sollicitude de la part de leurs proches se sentaient menacés de mort et dévalorisés respectivement dans 40,3% et 40,1%des cas, et enfin 41,2% d'entre eux se culpabilisaient. Tout ceci confirmait que les comportements de l'entourage influençaient le vécu des patients admis pour HDH.

**Table 3:** Relation entre comportements des accompagnants et leur représentation de la maladie

La représentation liée à la maladie	Patient considéré comme source de son mal (n/%)	Maladie incurable et/ou envoûtement (n/%)	Maladie naturelle (n/%)	TOTAL (n/%)
Comportements de l'entourage				
Abandon	27	66	5	98
	27,6%	67,3%	5,1%	100%
Agressivité	12	24	3	39
	30,8%	61,5%	7,7%	100%
Manque de sollicitude	28	62	5	95
	29,5%	65,3%	5,3%	100%
Sollicitude	1	8	8	17
	5,9%	47,1%	47,1%	100%
TOTAL	68	160	21	249
	27,3%	64,3%	8,4%	100%

C= 0,271 p < 0,0001 X^2^=36,86

**Table 4:** Relation entre le vécu du patient et les comportements de leurs accompagnants

Comportements de l'accompagnant	Abandon (n/%)	Agressivité (n/%)	Manque de sollicitude (n/%)	Sollicitude (n/%)	TOTAL (n/%)
Vécu du patient					
Menace de mort	76 42,0%	31 17,1%	73 40,3%	1 0,6%	181 100%
Dévalorisation	70 41,9%	30 18,0%	67 40,1%	0 0,0%	167 100%
Culpabilité	15 44,1%	4 11,8%	14 41,2%	1 2,9%	34 100%
Assurance	1 5,9%	0 0,0%	1 5,9%	15 88,2%	17 100%
TOTAL	162 40,6%	65 16,3%	155 38,8%	17 4,3%	399 100%

C= 0,62 p < 0,0001 X^2^=308,3

## Discussion

Cette étude est prospective et a porté sur 67 patients hospitalisés pour hémorragie digestive haute dans le service d'hépato-gastro-entérologie du CHU-Campus et 101 de leurs accompagnants. Nous avons eu avec les patients et leurs accompagnants un entretien semi-dirigé sur la base d'un guide d'entretien. L'analyse logico-sémantique des informations recueillies et l'inférence ont permis de comprendre les comportements des accompagnants dont les patients étaient admis pour HDH. Parfois, il fallait chercher à analyser les propos contradictoires du malade et des parents. Dans la présente étude, nous n'avons pas considéré le niveau d'instruction et la religion. Ces éléments constituent les limites de notre méthodologie. Mais, le fait que cette étude soit une des premières à aborder le problème de l'influence du comportement des accompagnants sur le vécu du malade au Togo, ces résultats méritent une attention particulière pour une meilleur prise en charge des malades en général et particulièrement ceux admis pour hémorragie digestive haute, pour qui la place singulière des familles dans l'accompagnement est primordiale [4]. Nous avions retrouvé parmi les accompagnants une prédominance féminine à 91,4%. Ceci concorde avec les résultats d'autres auteurs [5, 6] qui avaient trouvé une prédominance féminine à 65,4% chez les accompagnants ou aidants des patients.

La majorité de nos patients soit 67,2% des cas appartenaient à la classe socio-économique (CSE) basse; ceux issus des classes moyenne et élevé représentent respectivement 29,9% et 3%. Ces résultats concordent avec ceux de Agbebavi [7] qui avait trouvé que parmi les malades mentaux abandonnés à l'hôpital psychiatrique de Zébé, la majorité appartenait à la classe socio-économique (CSE) basse dans 52,17%. Ces résultats concordent avec ceux de Bouglouga et al [8] qui avaient montré dans leur étude sur le coût financier de la prise en charge hospitalière de la cirrhose et de ses complications, que le coût moyen d'une hémorragie digestive haute sur une durée moyenne de quinze jours d'hospitalisation était évalué à 519 euros. Ceci montre que la majorité des patients ayant une pathologie chronique vivent aussi dans la précarité. L'appartenance à une CSE basse de nos patients et de leurs familles fait que 83,2% des accompagnants avaient des difficultés financières et ainsi n'arrivent pas à faire face au coût lié à la prise en charge de l'HDH. Nos résultats étaient comparables à ceux d'autres auteurs [9, 10] qui avaient montré une inégalité d'accès au soins chez les personnes les plus vulnérables socialement en fin de vie ou présentant une pathologie chronique. Ces difficultés financières pouvaient être source de stress pour les accompagnants et leurs malades [10]; et pouvaient expliquer les comportements des accompagnants qui étaient marqués par l'abandon, le manque de sollicitude. Les difficultés financières, les conflits intrafamiliaux et les représentations négatives favorisaient l'abandon des patients admis pour HDH.

En effet, les comportements des accompagnants notamment, les accompagnants des patients hospitalisés pour HDH constituent encore de nos jours un domaine complexe et très peu exploré. Cependant, on sait que les facteurs socio-économiques et les facteurs psychologiques pouvaient influencer le comportement des accompagnants. Ainsi ces accompagnants, à cause de ces difficultés n’étaient pas à l’écoute ou ne répondaient pas aux besoins du patient et cela agissait sur le vécu du patient. La majorité de nos patients étaient des hommes et sont des sujets actifs (21 à 50 ans) c′est-à-dire l’âge qui correspond à la période de vie où l'individu devrait disposer de ses forces pour contribuer à la progression du groupe familial vers l'atteinte de ses objectifs. Mais lorsqu'il constitue plutôt un frein pour la famille dans la réalisation de ses tâches, ses chances s'amenuisent dans le groupe. Dans notre étude, la majorité de nos patients présentait une maladie chronique et ils se plaignaient souvent du manque d'attention de la part de leurs accompagnants. Ce résultat était contraire à celui de Bogalska et al [11] qui avaient montré que pour les accompagnants la peur de souffrir ou de voir le patient souffrir était une forme partagée de la souffrance du malade. Cette réclamation d'attention dans notre étude pouvait être liée à la régression du patient qui est une fuite psychologique devant l'angoisse de mort provoquée par la maladie, le malade peut aussi présenter une dépendance et dans cet état il attend que son entourage s'occupe de lui sur le plan physique que moral. En effet, le caractère chronique de la maladie demandait beaucoup de la part de l'entourage et on observait alors un déphasage entre les demandes du patient et les préoccupations de la famille. Ceci est conforme aux données de la littérature [11–13] ou il est noté que face à des maladies chroniques, les personnes malades, les accompagnants et les professionnels de santé développent des logiques d'action qui ne peuvent pas être parfaitement concordantes. Chaque partie se réfère à des règles et détermine des ordres de priorités qui peuvent engendrer des tensions ou des conflits. Les difficultés financières, les conflits intra familiaux préexistants ou ceux que la maladie occasionne, ont un impact sur la cohésion du groupe familial et remettent ainsi en cause la dynamique familiale consciente ou inconsciente. L'HDH qui survient souvent au décours d'une maladie chronique vient donc amplifier ces difficultés et traumatise davantage ces familles. Les conflits intrafamiliaux permanents y trouveront parfois leur origine. Ces conflits étant angoissants, la famille est à la recherche permanente de défenses pour s'en sortir. Ces défenses peuvent être adaptées ou non. Dans le cas précis des familles qui finissent par abandonner le malade, les défenses longtemps adoptées pour réguler l’équilibre du système et surmonter l'adversité, s'affaiblissent et les modifications qu'entraîne la situation du malade sont finalement perçues comme destructrices et traumatisantes. Les défenses adoptées par ces familles ne semblent plus adaptées à la réalité sociale. Pour ces familles, la solution pour sortir de ce traumatisme ne sera plus que de se débarrasser du malade qui en est la cause. La souplesse et la tolérance font place à la rigidité et l'intolérance. De plus, les représentations que les parents ont de la maladie dont souffre le sujet, fragilisent le lien entre ce dernier et la famille, surtout qu'elles sont négatives c′est-à-dire que le patient considéré comme source de sa maladie, maladie incurable, envoûtement et/ou empoisonnement [14]. Ces représentations sont relatives aux croyances et traditions de la famille, mais aussi aux préjugés et à toutes sortes de légendes anciennes mais toujours vivaces à propos des maladies chroniques. Dans notre étude, ces représentations sont en général conformes à la représentation culturelle négro-africaine de la dynamique des forces de l'univers [15]. Dans cette dynamique, rien ne survient de façon fortuite; on trouve toujours une cause qui sous-tend l'apparition des désordres de tout genre. La maladie est perçue comme la rupture du malade avec le monde dont il dépend (conséquence de ses actes), d'où le sentiment de rejet et de dévalorisation aggravé par les croyances en un châtiment émanant des ancêtres, des génies ou des autres membres de la société [15].

Ainsi, les représentations permettent de donner du sens à certaines particularités de la dynamique familiale. Les préjugés au sujet de la maladie chronique créent donc un cercle vicieux d'ostracisme et de discrimination qui conduit à l'isolement social du malade. Le fort pourcentage (67,2%) des familles de la CSE basse révèle que les difficultés financières peuvent contraindre certaines familles à abandonner le malade chronique. Toutefois, si on peut constater que, des familles de la CSE élevée abandonnent les malades admis pour HDH, alors, cette raison parait insuffisante pour justifier l'abandon du malade. Ces clivages dans la famille témoignent les difficultés relationnelles au sein de ces familles. Divisée par les conflits intrafamiliaux, la famille ne peut plus véritablement s'unir autour du malade. Ainsi dans notre étude, la charge (rester auprès du malade, payer les médicaments, faire à manger, laver les habits, laver le patient) de s'occuper du malade est souvent confié à une femme qui était seule à tout faire. Le plus souvent, elles étaient animées d'un sentiment de fatigue et d'un sentiment d’être seules à s'occuper du malade même si les frais du traitement étaint souvent honorés par d'autres membres de la famille. En plus du caractère chronique de la maladie ces accompagnants finissaient souvent par s’écarter du malade qui se sentait abandonné. Parmi ces accompagnants nous avions des parents proches et parents éloignés du patient mais nous avions pu relever aussi des accompagnants qui étaient des inconnus du malade. C’était des gens que la famille payait pour rester auprès du malade. Ce phénomène favorisait le sentiment du patient d’être rejeté, dévalorisé ou humilié par les siens. Ces comportements témoignent donc de l'existence des difficultés relationnelles dans la famille. Divisée par des conflits intrafamiliaux la famille ne pouvait plus s'unir autour du malade. Dans la dynamique familiale, le patient rendu invalide par la maladie et ne pouvant plus répondre aux attentes et aux aspirations de la famille, devenait plutôt une charge. Sa présence dans la famille devenait angoissante, ses chances à être abandonné s'accroissaient, d'autant plus qu'il était une source de dépense énorme pour la famille. Il constituait ainsi dans la famille un pôle émetteur d’énergie négative agissant sur la cohésion du groupe à cause des difficultés et des conflits que sa situation engendrait. Mais certaines familles s'adaptaient à l’événement et éprouvaient une certaine satisfaction à répondre aux difficultés et aux nécessités de l'accompagnement du malade en fin de vie [6]. En tant que système vivant, la famille s'autorégule; elle doit s'adapter aux nécessaires changements des différents moments de son organisation ainsi qu'aux changements de la société. Pour ce faire, la famille doit pouvoir faire preuve de souplesse et de tolérance face aux modifications qu'entraînent les changements en cas de maladie.

Il s'agit de la résilience familiale qui correspond à la capacité des membres de la famille à faire émerger des aspects de soi résolument positifs dans l'adversité. Ainsi les pires moments peuvent devenir les meilleurs. La dimension des croyances familiales était également utile pour comprendre la résilience. La signification qu'une famille attribue à l’événement problématique est déterminante dans la manière dont elle va surmonter l'adversité. On comprend donc pourquoi des familles ayant des représentations négatives de l'HDH pouvaient abandonner le malade ou avoir des comportements d'agressivité verbale ou physique envers lui. Dans la présente étude, l'expérience des accompagnants dans l'accompagnement des malades jouait un rôle très important dans les comportements des accompagnants. Ainsi, ceux qui avaient une fois ou plusieurs fois été au chevet d'un malade réagissaient différemment que ceux qui vivaient cette expérience pour la première fois. Les résultats montraient donc que plus on avait une expérience dans l'accompagnement des malades en milieu hospitalier, plus on développait des mécanismes de tolérance et d'adaptation vis-à-vis des attitudes régressives et infantiles des patients. Par conséquent les difficultés qu'engendraient la maladie chronique au rang desquels l'HDH, les représentations négatives et des défaillances dans le fonctionnement du système familial, rendant la famille incapable à surmonter longtemps l'adversité liée à la maladie chronique, peuvent contraindre des familles à adopter des comportements inadaptés face à un patient et cela agira négativement sur le vécu de ce dernier.

## Conclusion

Cette étude nous a permis d'atteindre nos objectifs scientifiques car nous avons pu montrer que les facteurs socio-économiques et les facteurs psychologiques influencent les comportements des accompagnants qui à leur tour avaient un impact sur le vécu du patient admis pour hémorragie digestive haute dans notre service. Pour ce qui est des objectifs d'application, leur atteinte dépendra de la vulgarisation des résultats de cette recherche.
